# Angiotensin Type-1 Receptor Blockade May Not Protect Kidney against Cisplatin-Induced Nephrotoxicity in Rats

**DOI:** 10.1155/2014/479645

**Published:** 2014-03-16

**Authors:** Roya Rastghalam, Mehdi Nematbakhsh, Mehrnoosh Bahadorani, Fatemeh Eshraghi-Jazi, Ardeshir Talebi, Maryam Moeini, Farzaneh Ashrafi, Soheila Shirdavani

**Affiliations:** ^1^Water and Electrolytes Research Center, Isfahan University of Medical Sciences, Isfahan 81745, Iran; ^2^Department of Biology, Falavarjan Branch, Islamic Azad University, Isfahan 84515, Iran; ^3^Department of Physiology, Isfahan University of Medical Sciences, Isfahan 81745, Iran; ^4^Isfahan MN Institute of Basic and Applied Sciences Research, Isfahan 81546, Iran; ^5^Department of Clinical Pathology, Isfahan University of Medical Sciences, Isfahan 81745, Iran; ^6^Department of Internal Medicine, Isfahan University of Medical Sciences, Isfahan 81745, Iran

## Abstract

*Background*. Cisplatin (CDDP) is an anticancer drug, which is accompanied with major side effects including nephrotoxicity. We tested two doses of losartan (10 and 20 mg/kg/day) against nephrotoxicity in a rat model treated with daily administration of CDDP (2.5 mg/kg/day). *Methods*. Five groups of rats were examined. Groups 1 and 2 received losartan 10 and 20 mg/kg/day, i.p, for a period of 10 days. Group 3 received saline for 10 days, but from day 3 the animals received CDDP (2.5 mg/kg/day, i.p) for the next seven days. Groups 4 and 5 received treatment regimen the same as groups 1 and 2, but from day 3 they also received CDDP for the next seven days. At the end of the experiment, blood samples were obtained and the kidneys were removed to undergo pathological investigation and to obtain supernatant from homogenized tissue. *Results*. CDDP induced nephrotoxicity, but the serum levels of creatinine and blood urea nitrogen were not attenuated by losartan. The pathological findings confirmed that losartan did not have nephroprotective effect in this experimental model.* Conclusion*. According to the findings, losartan could not improve renal function impaired by toxicity induced by continuous doses of CDDP, and also it worsened the renal failure.

## 1. Introduction

Cisplatin or cis-diamminedichloroplatinum (II) (CDDP) is an anticancer drug for treatment of solid tumors [1, 2]. One of the major side effects of CDDP is nephrotoxicity, which is known as a limitation for CDDP therapy in clinic [3]. CDDP is removed by the kidney by both glomerular filtration and tubular secretion [4]. Inflammation, oxidative stress, and change in the renal circulation may also be induced by CDDP [4–6]. To decline CDDP-induced nephrotoxicity, recent studies have concentrated on many supplementations [7–11]. Losartan is an angiotensin type-1 receptor blocker as well as an antioxidant, which has been suggested to be nephroprotective against CDDP-induced nephrotoxicity by others [10, 11]. Losartan may reduce renal disease progression in patients with type 2 diabetes [12] and decrease hypertension [13] and microalbuminuria or proteinuria [14, 15]. Several studies have presented different effects of losartan on nephrotoxicity induced by nephrotoxic compounds such as CDDP [10, 11, 16, 17]. Losartan may prevent nephrotoxicity caused by administration of single dose of CDDP in males [11, 16, 17], but it promotes renal damage in females [11]. Although the nephroprotective role of losartan against single dose of CDDP was reported in studies, CDDP is clinically administered sequentially. Therefore, we designed the current study to investigate the effect of losartan on renal toxicity induced by sequential (daily) infusion of CDDP.

## 2. Materials and Methods

### 2.1. Animals

Forty-two male Wistar rats (Animal Centre, Isfahan University of Medical Sciences) weighing 175.56 ± 2.24 g were used. Animals were kept under standard conditions. Food and water were provided freely. This research was approved in advance by the Isfahan University of Medical Sciences Ethics Committee.

### 2.2. Experimental Protocol

The animals were divided into five groups. Groups 1 and 2, as the negative control groups, received intraperitoneal (IP) losartan 10 and 20 mg/kg/day for a period of 10 days, respectively. Group 3, as the positive control group, received saline for a period of 10 days, but from day 3 the animals received CDDP (2.5 mg/kg/day, IP) for the next seven days. Groups 4 and 5 received treatment regimen the same as groups 1 and 2, respectively, but from day 3 they received CDDP for the next seven days. The animals' body weight was recorded on a daily basis. At the end of the experiment, all animals were anesthetized by ketamine (75 mg/kg) to obtain blood samples via heart puncture. The serum was kept in −20 until measurement. Finally, the animals were sacrificed; kidneys were removed and immediately weighed. The left kidney was fixed in formalin for pathological investigations, and the right kidney was homogenized and centrifuged at 15000 g and the supernatant was kept in −20 until measurement.

### 2.3. Measurement

The levels of serum creatinine (Cr), blood urea nitrogen (BUN), total proteins (TP), magnesium (Mg), and lactate dehydrogenase (LDH) were measured using diagnostic kits (Pars Azmoon, Tehran, Iran). The serum and kidney levels of nitrite (stable nitric oxide metabolite) were measured using ELIZA kit (Promega Corporation, Madison, WI, USA). The serum and kidney levels of malondialdehyde (MDA) were measured by a manual method. Briefly, 0.5 cc of the sample was added to 1 cc of trichloroacetic acid 10%. This mixture was centrifuged at 2000 g for 10 minutes. Then, 0.5 cc of the supernatant was mixed with 0.5 cc of thiobarbituric acid 0.67% and placed in the boiling water for 10 minutes. After cooling, the absorbance was determined at the wavelength of 532 nm.

### 2.4. Histopathological Procedures

The left kidney was fixed in neutral formalin 10% and embedded in paraffin. After slicing, hematoxylin and eosin staining was performed to examine the samples for tubular atrophy, as well as the presence of casts, debris, and necrotic materials in the tubular lumen. Intensity of the tubular lesion was scored from 1 to 4, while zero was assigned to the normal tissue without damage.

### 2.5. Statistical Analysis

Data were reported as mean ± SEM. The levels of BUN, Cr, LDH, MDA, TP, Mg, nitrite, and body and kidney weights were analyzed by the one-way ANOVA followed by the LSD test. Tissue damage scores were compared by the Kruskal-Wallis or Mann-Whitney tests. *P* values less than 0.05 were considered statistically significant.

## 3. Results

### 3.1. Effect of CDDP and Losartan on Serum BUN and Cr Levels

**Figure 1:**
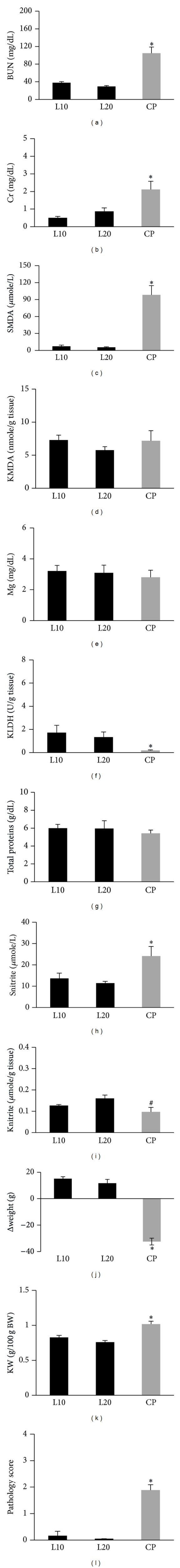
Comparison of the measured biochemistry parameters in serum and kidney tissue, kidney damage score, kidney weight, and body weight between the negative (treated with losartan alone) and positive (treated with CP alone) control groups. Data is reported as mean ± SEM. *indicates significant difference from the negative control groups, and ^#^indicates significant difference from L20 group (*P* < 0.05). BUN: blood urea nitrogen, Cr: creatinine, SMDA: serum malondialdehyde, Mg: magnesium, Snitrite: serum nitrite, KMDA: kidney MDA, Knitrite: kidney nitrite, KW: kidney weight per 100 g bodyweight, L10 and L20: losartan treated groups with dose of 10 and 20 mg/kg, respectively, and CP: cisplatin.

**Figure 2:**
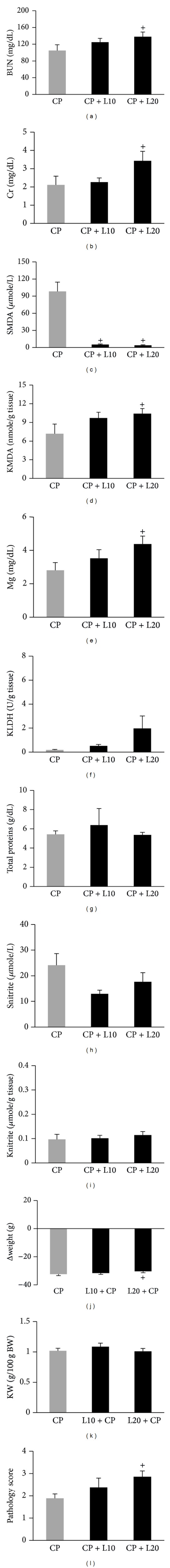
Comparison of the measured biochemistry parameters in serum and kidney tissue, kidney damage score, kidney weight, and body weight between positive control group (treated with CP alone) and losartan with CP treated groups. Data is reported as mean ± SEM. ^+^indicates significant difference from the positive control group (*P* < 0.05). BUN: blood urea nitrogen, Cr: creatinine, SMDA: serum malondialdehyde, Mg: magnesium, Snitrite: serum nitrite, KMDA: kidney MDA, Knitrite: kidney nitrite, KW: kidney weight per 100 g bodyweight, L10 and 20: losartan treated groups with dose of 10 and 20 mg/kg, respectively, and CP: cisplatin.

The results revealed that nephrotoxicity was induced by CDDP, which was characterized by significant increase in the serum levels of BUN and Cr (*P* < 0.05). Administration of losartan at the doses of 10 and 20 mg/kg did not alter the serum levels of BUN and Cr, while coadministration of CDDP and losartan elevated the serum levels (10 mg/kg, insignificantly, and 20 mg/kg, significantly, *P* < 0.05), when compared with the CDDP alone (positive control) group (Figures 1 and 2).

### 3.2. Effect of CDDP and Losartan on Serum and Kidney MDA Levels

CDDP significantly elevated the serum level of MDA (*P* < 0.05) when compared with the negative control groups. However, administration of losartan (10 and 20 mg/kg) accompanied with CDDP attenuated the serum level of MDA when compared with the CDDP alone treated group (positive control group) (*P* < 0.05). Considering kidney tissue MDA level, no significant difference was observed between the losartan alone-treated groups (10 and 20 mg/kg) and the CDDP alone treated group (*P* < 0.05). However, coadministration of losartan (20 mg/kg) and CDDP significantly increased the kidney tissue level of MDA (*P* < 0.05) when compared with the CDDP alone treated group (Figures 1 and 2).

### 3.3. Effect of CDDP and Losartan on Serum and Kidney Nitrite Levels

The serum level of nitrite in the positive control group significantly increased compared with the negative control groups (*P* < 0.05), while coadministration of CDDP and losartan (10 and 20 mg/kg) decreased the serum level of nitrite insignificantly. The kidney tissue level of nitrite was decreased by CDDP alone, and combination of losartan (either 10 mg/kg or 20 mg/kg) and CDDP did not alter the results (Figures 1 and 2).

### 3.4. Effect of CDDP and Losartan on Kidney LDH Level

Tissue level of LDH in the positive control group was significantly decreased when compared with the negative control groups (*P* < 0.05). However, coadministration of losartan (10 and 20 mg/kg) and CDDP compared to administration of CDDP alone increased the tissue level of LDH insignificantly (Figures 1 and 2).

### 3.5. Effect of CDDP and Losartan on Mg and TP Levels

No significant difference in the TP serum level was observed between the groups. The serum level of Mg was significantly increased by CDDP plus losartan (20 mg/kg) when compared with the positive control group (*P* < 0.05, Figures 1 and 2).

### 3.6. Effect of CDDP and Losartan on Body and Kidney Weight

CDDP reduced bodyweight. Administration of 20 mg/kg losartan accompanied with CDDP significantly ameliorated weight loss (*P* < 0.05). CDDP also increased kidney weight significantly (*P* < 0.05), while coadministration of losartan (either 10 mg/kg or 20 mg/kg) and CDDP did not alter the results (Figures 1 and 2).

### 3.7. Effect of CDDP and Losartan on Kidney Damage

**Figure 3:**
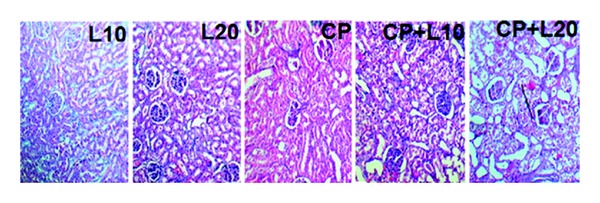
The sample images of kidney tissue stained with H&E to examine tissue damage in the kidney of five experimental groups. L10, L20, CP, CP+L10, and CP+L20 indicate the samples from groups treated with losartan (10 mg/kg), losartan (20 mg/kg), CP, CP plus low dose of losartan, and CP plus high dose of losartan, respectively. Higher damage scores were observed in the groups treated with cisplatin plus losartan.

The kidney tissue obtained from the negative control groups was considered normal. CDDP induced kidney damage when compared with the negative control groups (*P* < 0.05  Figures 1 and 2). In addition, CDDP accompanied with losartan (10 and 20 mg/kg) intensified the damage. High dose of losartan plus CDDP provided significant increase in kidney tissue damage when compared with the CDDP alone treated group (*P* < 0.05) (Figures 2 and 3).

## 4. Discussion

In the current study, we attempted to investigate the effect of losartan on renal toxicity induced by daily administration of CDDP. We found that losartan is not a nephroprotectant agent against CDDP nephrotoxicity in this rat model. CDDP affects kidney and body weight; serum Cr, BUN, TP, osmolality, and nitric oxide levels [18, 19] and previous reports indicated that losartan is a nephroprotectant agent against CDDP-induced nephrotoxicity [10, 11, 16]. Saleh et al. reported administration of single dose of CDDP and losartan reduced kidney toxicity [16]. In our pervious studies, we also administered single dose of CDDP and found that losartan is nephroprotectant in males [10, 11] but not in females [11, 20]. In contrast, Deegan et al. reported that injection of low and high single doses of losartan (10 and 30 mg/kg) two hours prior to CDDP injection could not ameliorate nephrotoxicity induced by CDDP [17]. This is while Azzadin et al. demonstrated that the losartan increased cyclosporine-induced nephrotoxicity in rat [21]. Our results are consistent with their findings. Also, Poormoosavi et al. reported that losartan intensifies nephrotoxicity induced by gentamicin, while coadministration of cimetidine (as a cytochrome-P450 inhibitor) and losartan could decrease the nephrotoxicity induced [22]. Losartan also has inconsistent effects on renal function [23] and induces renal vasodilation, inhibits reduction of glomerular filtration rate in hypertension, decreases proteinuria, and reduces morbidity and mortality in diabetic nephropathy [23, 24].

As mentioned above, we observed that administration of losartan promotes renal dysfunction induced by CDDP, which is shown by increasing the values of BUN, Cr, and kidney tissue damage score. Losartan increases renal blood flow [25] that may accumulate more CPPD in the kidney. In addition, it seems that continuous or single dose administration of CDDP may induce different levels of kidney tissue damage. In the present study, the animals received CDDP in total amount of 17.5 mg/kg (more than twice of single dose of CDDP in our previous study [11]). Therefore, the cumulative dose of CDDP increased in the kidneys and enhanced renal toxicity.

CDDP reduced body weight probably due to gastrointestinal disturbances. This finding is in agreement with those reported in other studies [26, 27]. High dose of losartan (20 mg/kg) ameliorated the bodyweight loss. Losartan affects aldosterone secretion [28], and this may lead to retention of water and electrolytes to prevent the weight loss induced by CDDP. Kidney weight was also increased by CDDP that could be related to retention of water and solutes in renal tissues or changes induced by CDDP in renal blood flow.

Our results showed that administration of CDDP increased serum level of MDA as a final product of lipid peroxidation and administration of losartan (10 and 20 mg/kg) as an antioxidant could ameliorate this. It is documented that losartan prevents lipid peroxidation in renal tubules [29]. It seems that losartan can reduce the production of oxygen free radicals and inhibit the oxidative pathway. An evidence showed that Telmisartan, an angiotensin II receptor antagonist (angiotensin receptor blocker, ARB), can inhibit the production of oxygen free radicals and improve renal function [30].

In the current study, it was also observed that CDDP increased the serum level of nitrite and decreased kidney tissue level of nitrite. NO, as a vital agent, acts via various mechanisms. CDDP increases iNOS and reduces eNOS. iNOS can produce NO in the body more than eNOS and nNOS [31–33]. Possibly, CDDP increased serum level of iNOS and decreased renal level of eNOS.

Our results showed that administration of CDDP alone did not change the serum levels of Mg and TP. It is documented that serum level of Mg decreased in the third week after CDDP administration [34]. Proximal tubule is the main site of protein reabsorption and CDDP degenerates this structure and subsequently protein uptake is reduced and protein urinary excretion is increased [4]. Therefore, our observations associated with Mg and TP may be related to the experiment protocol.

## 5. Conclusion

The supportive role of losartan in preventing cardiovascular disease [35] or even reducing nephrotoxicity induced by CDDP [10, 11, 16] has been reported previously. However, in the current study, it was found that losartan could not improve renal toxicity induced by continuous administration of CDDP and also intensified renal failure. The pathological findings confirmed these observations. Carrying out further investigations is necessary to investigate the combination of losartan and other agents.
